# Infant Feeding Policy and Programming During the 2014–2015 Ebola Virus Disease Outbreak in Sierra Leone

**DOI:** 10.9745/GHSP-D-16-00387

**Published:** 2017-09-27

**Authors:** Amelia Brandt, Óscar Serrano Oria, Mustapha Kallon, Alessandra N. Bazzano

**Affiliations:** a Tulane University School of Public Health and Tropical Medicine, New Orleans, LA, USA.; b Humanitarian Nutrition, Freelance Specialist, London, UK.; c GOAL Sierra Leone, Freetown, Sierra Leone.

## Abstract

Optimal breastfeeding is of vital importance to infant and child health and has been adopted by countries as a standard recommendation. However, in the context of an infectious disease outbreak, especially when the disease is poorly understood, policy makers must balance the benefits of breastfeeding against the risk of disease transmission through breastfeeding. During the 2014–2015 Ebola Virus Disease (EVD) outbreak in Sierra Leone, the development of correct and consistent messaging about infant feeding and nutrition programming was considerably delayed by numerous challenges. These challenges included a lack of sufficient information about the risk of transmission through human milk, numerous stakeholders, limited communication between coordination pillars, inconsistent and evolving messages from various stakeholders, and the public's distrust of the health system and international actors. For improved response to future disease outbreaks, research on vertical transmission of EVD should be prioritized, infant and young child feeding experts should be integrated into outbreak response collaboration, and a digital repository of potential and appropriately tailored messages should be created.

## Abstract (precis)

Policies on breastfeeding and possible mother-to-child transmission of Ebola Virus Disease (EVD) during the outbreak evolved depending on public health priorities and the evidence available at that particular time. To improve responses to future outbreaks, research on vertical transmission of EVD should be prioritized; infant and young child feeding experts should be integrated into the outbreak response; and a digital repository of national policies and associated messages should be created.

## BACKGROUND

The 2014–2015 Ebola Virus Disease (EVD) outbreak in West Africa was unprecedented in its severity. In Sierra Leone, the first case was recorded in May 2014, and the outbreak continued for nearly 18 months until the country was declared Ebola-free by the World Health Organization (WHO) on November 7, 2015.^1^^,^^2^ During that time, 14,124 total cases were recorded in the country.^1^ Difficulties in developing infant feeding policy and programming during this outbreak closely mirror those of the early years of the HIV epidemic, albeit with a greatly accelerated timeline. In this article, we provide an overview of the infant feeding policies and programming that evolved during the 2014–2015 EVD outbreak in Sierra Leone.

EVD, previously referred to as Ebola hemorrhagic fever, is a rare disease caused by contact with 1 of the 5 species of Ebola virus.^3^ EVD was first discovered in 1976 in what is now the Democratic Republic of the Congo. Since that time, Africa has experienced small sporadic outbreaks,^3^ the largest of which took place in Gulu, Uganda, in 2000–2001 with 425 cases. The 2014–2015 outbreak was by far the most severe in history.^4^

In humans, the virus spreads through “direct contact (through broken skin or mucous membranes) with the blood, secretions, organs, or other bodily fluids” of a person who is sick with or has died from EVD.^5^ As such, public health professionals recognized that there was a feasible risk of transmission between a breastfeeding mother with EVD and her uninfected infant through both breast milk and close contact with other bodily fluids during breastfeeding.

The risk of transmission through breastfeeding for mothers who had survived EVD was difficult to estimate, although a number of studies are currently seeking to increase that understanding. At the start of the outbreak, what evidence was available indicated that the Ebola virus could persist in some bodily fluids in the convalescent stage. This viral persistence was thought to be due to the fact that certain organs of the body, such as the testes and mammary glands, are immunologically privileged—meaning, that they are able to tolerate disease antigens without causing an immune response.^6^ While several studies focused on this viral persistence, only 1 tested human milk for Ebola virus during convalescence. The sample was found positive for the virus using both reverse transcription polymerase chain reaction (RT-PCR) and viral culture 15 days after disease onset.^6^^,^^7^ In contrast, the virus had been isolated from semen samples up to 82 days following disease onset.^8^ The identification of virus in human milk by RT-PCR, however, was not necessarily indicative of infectivity,^9^ which further complicated the interpretation of the existing evidence.

**Figure:**
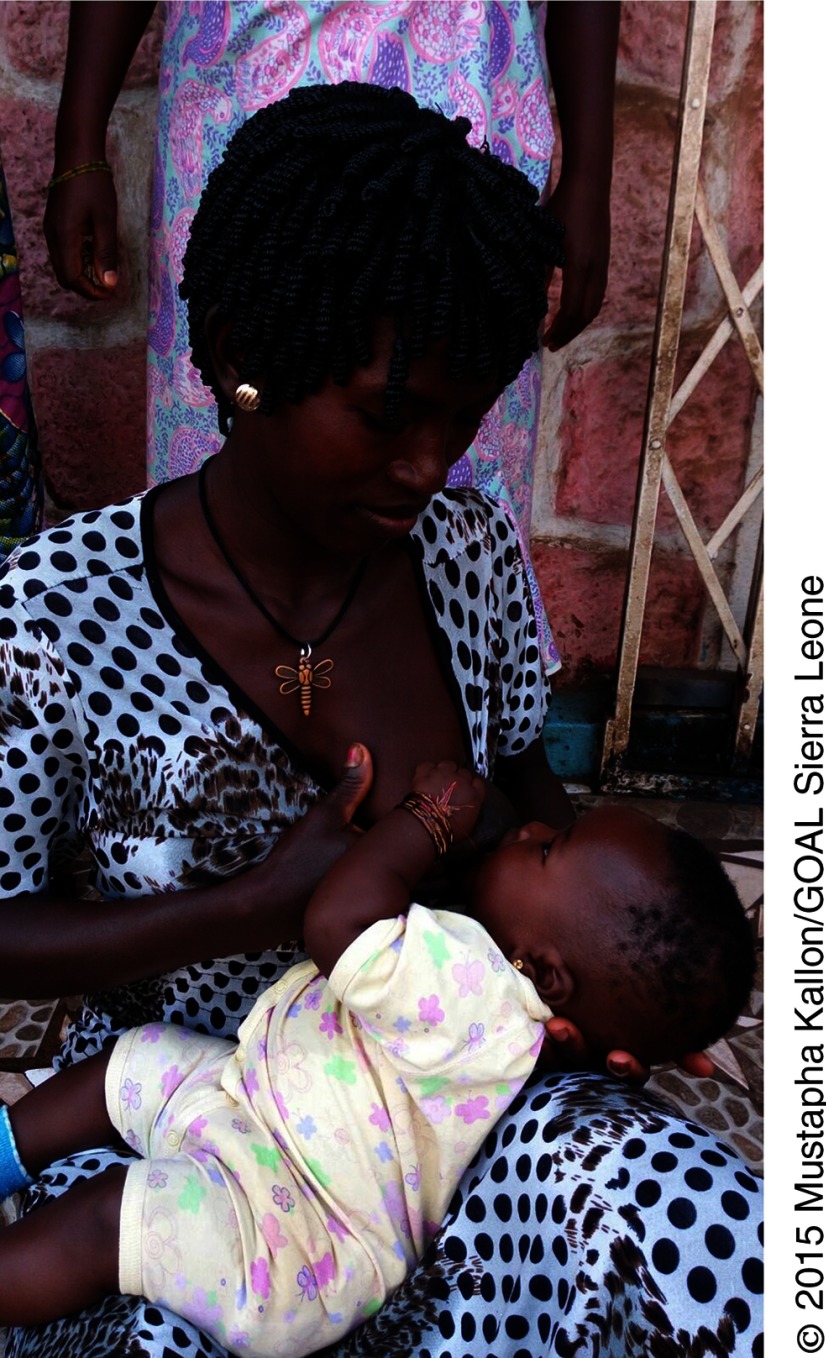
A woman in Sierra Leone breastfeeds her infant.

In this context, the potential risk of EVD transmission through breastfeeding had to be balanced with the benefits of optimal breastfeeding. WHO-recommended breastfeeding practices include initiating breastfeeding within 1 hour of birth, “exclusive breastfeeding for the first 6 months of life, and introduction of nutritionally adequate and safe complementary (solid) foods at 6 months together with continued breastfeeding up to 2 years of age or beyond.”^10^ It is estimated that optimal breastfeeding could avert 800,000 deaths in children under 5 years old, although globally only 36% of infants from 0 to 6 months are exclusively breastfed.^10^^,^^11^

## COUNTRY CONTEXT

Sierra Leone has one of the highest under-5 mortality rates in the world, which makes achieving optimal breastfeeding especially important.^12^ The Sierra Leone Ministry of Health and Sanitation (MOHS) demonstrated a commitment to breastfeeding promotion prior to the outbreak, by launching a comprehensive nutrition program in 2009 with Infant and Young Child Feeding (IYCF) components that strongly encouraged breastfeeding.^13^ This program had contributed to increases in recommended feeding practices within its first several years. In 2013, exclusive breastfeeding of children from birth to 6 months had increased from 11% to 32% within that period, and 97% of children were estimated to have been breastfed at some point.^14^^,^^15^

From 2009 to 2013, exclusive breastfeeding of children from birth to 6 months increased from 11% to 32%.

These gains in optimal breastfeeding coincided with a small reduction in under-5 mortality. In the 5-year period preceding the 2008 Demographic and Health Survey (DHS), under-5 mortality was estimated at 156 deaths per 1,000 live births.^14^ In comparison, the rate for the 5-year period preceding the 2013 DHS was 140, a reduction of approximately 10%.^15^ During and after the EVD outbreak, the MOHS and implementing partners were concerned that policies, messaging, and programming about infant feeding for women currently or previously infected with EVD could cause a reduction in optimal breastfeeding among uninfected women. This conflicted with the broad recognition that it was crucial to take every measure to prevent EVD transmission, especially among the most vulnerable.

## STAKEHOLDERS

A number of health and nutrition organizations active in Sierra Leone during the outbreak were involved in the development of policies, messaging, and programming related to infant feeding within the context of the EVD outbreak. These stakeholders included the MOHS, WHO, the United Nations Children's Fund (UNICEF), the U.S. Centers for Disease Control and Prevention (CDC), the Emergency Nutrition Network (ENN), and numerous local, national, and international civil society organizations. While civil society organizations were not directly responsible for policy making, they contributed considerable technical expertise and experience.

The office of the President of Sierra Leone created the National Ebola Response Centre and District Ebola Response Centres to coordinate the outbreak response and a pillar system to facilitate coordination within specific sectors: child protection and psychosocial, case management, communications, logistics, safe burials, social mobilization, surveillance, coordination, and food security.^16^

## POLICY AND MESSAGING

The Social Mobilization Pillar was responsible for developing and maintaining the *Consolidated Messaging Guide for Ebola Communication in Sierra Leone* (henceforth the *Consolidated Messaging Guide*) of approved messages, which was frequently revised as new information became available. While the Social Mobilization Pillar produced a majority of the health messaging, it is important to note that other pillars also created health communication tools specific to their sector. For example, the Psychosocial Support Pillar was responsible for the majority of survivor issues and created health communication tools for survivors, although messaging for survivors was also included in the *Consolidated Messaging Guide.* A formal mechanism to coordinate messages between the pillars did not exist, although informal coordination did occur.

### Policy and Messaging Within Ebola Treatment Centres

To address infant feeding in the EVD context, guidance was developed for both women with acute EVD and those in convalescence (survivors). Initial breastfeeding guidance was issued on August 22, 2014, by the ENN through a consultation involving in-country staff and experts from the CDC, the Liberia Ministry of Health and Social Welfare, UNICEF, and WHO.^17^ Although this guidance was used in Sierra Leone, Liberia was included in this initial consultation because of their experience addressing the outbreak. This guidance advised the cessation of breastfeeding for mothers with EVD in all cases, except when both mother and child were EVD positive.^17^ Mothers who were EVD positive, but whose infants were EVD negative, were advised to stop breastfeeding as the risk of infection through continued breastfeeding outweighed the risks of replacement feeding.^17^ These recommendations remained consistent when the guidance was updated in September 2014.^18^ Although the guidance was incorporated into national technical guidance designed for use by professional stakeholders, as far as the authors know, it was not integrated into public messaging.

Several actors involved in the treatment of EVD patients argued that continuing breastfeeding would increase the already high mortality risk of patients and, as such, implemented total cessation of breastfeeding, even if both mother and infant were EVD positive. This approach became the final recommendation for Ebola Treatment Centres (ETCs) until the end of the epidemic, although “the rationale was based on anecdotal cases, limited field experience and the assumption that the presence of Ebola virus in breast milk increases the likelihood of severe Ebola in an already infected infant.”^19^ In these cases, ready-to-use infant formula (RUIF) was provided to replace human milk and administered by either ETC staff or the mother using the standard infection prevention and control procedures of the ETC. No further evidence was produced to argue for a less conservative approach. However, the same guidelines provided that the infected mother of an infected child must be given the choice of breastfeeding, if she prefers, as long as she was properly informed and advised.^18^

Ready-to-use infant formula was provided to replace human milk and administered by either ETC staff or the mother using the standard infection prevention and control procedures.

### Messaging and Policy for Survivor Women in Convalescence

The first messages regarding breastfeeding during convalescence were included in the August 2014 ENN memo.^17^ The memo recommended the cessation of breastfeeding by survivor mothers until their breast milk tested negative for the Ebola virus.^17^ This guidance was updated in September 2014 to include WHO recommendations that when testing was not feasible, mothers could resume breastfeeding after 8 weeks.^18^ Expressing milk was recommended only to alleviate pain for the mother, although this guidance was not made clear to survivors in any standard way.

**FIGURE 1:**
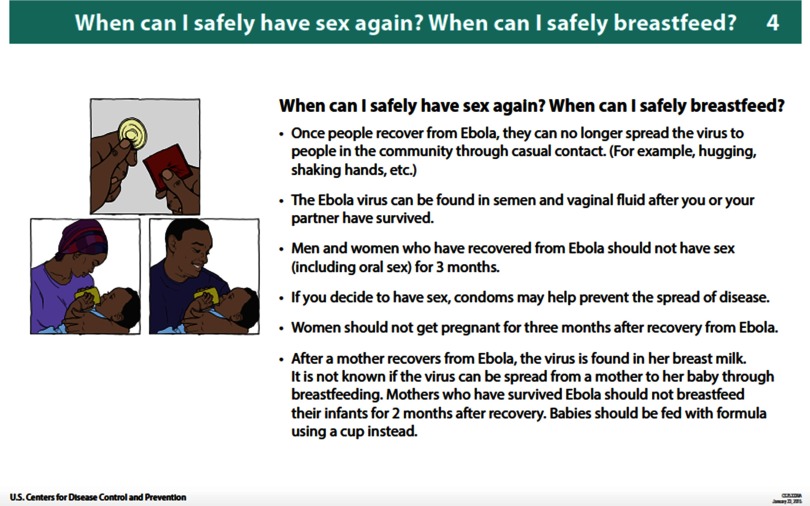
Initial CDC EVD Survivor Information Book Recommending Cessation of Breastfeeding by Survivors Until 2 Months Post-Recovery, January 2015 Abbreviations: CDC, U.S. Centers for Disease Control and Prevention; EVD, Ebola virus disease. Note: This guidance was retracted and corrected after new information about breastfeeding risk emerged.

In January 2015, the CDC produced an information book for survivors recommending the cessation of breastfeeding by survivors until 2 months following recovery (Figure 1).^20^ In March 2015, the Social Mobilization Pillar published the following messaging in the *Consolidated Messaging Guide* that was inconsistent with CDC's information book^21^:

If you have survived Ebola, it is best not to breastfeed IF you have other safe ways to feed your baby. But if there is no other way to feed your baby safely, breastfeeding will still provide the nutrition your baby needs.

The discrepancy in the existing messages was brought to the attention of the Social Mobilization Pillar, MOHS, CDC, and WHO in March 2015.

**FIGURE 2:**
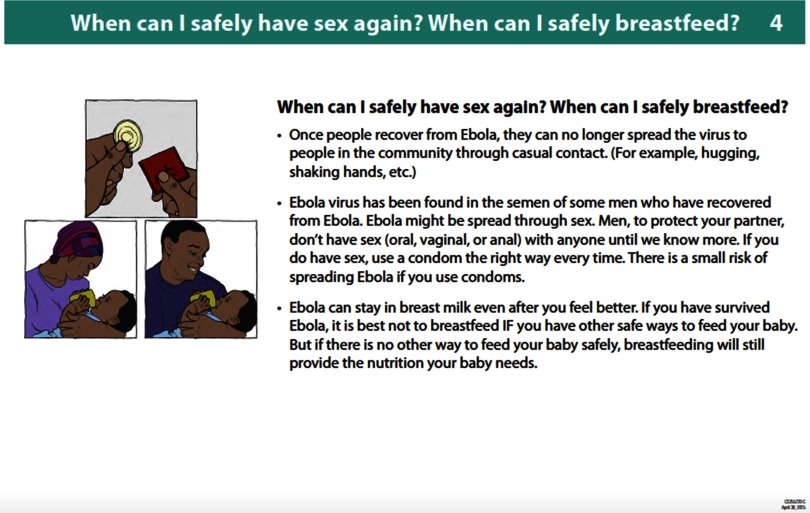
Updated CDC EVD Survivor Information Book Recommending Avoiding Breastfeeding by Survivors if Other Safe Feeding Methods Are Available, April 2015 Abbreviations: CDC, U.S. Centers for Disease Control and Prevention; EVD, Ebola virus disease.

After an examination of the existing messages and the available information regarding risk to infants, all parties agreed to adopt the messaging found in the *Consolidated Messaging Guide*. In April 2015, the CDC revised their survivor information book to make the messaging consistent (Figure 2).^22^ The earlier technical guidance from ENN had not been distributed to the Sierra Leonean public, and as such, official revisions were not made. Given the existence of conflicting messages at the policy and messaging level, it is likely that women were also given conflicting messages at the individual level, but this was not documented.

On April 11, 2016, WHO issued interim guidance on clinical care for EVD survivors that addressed breastfeeding.^23^ This guidance was the most permissive, stating that lactating women may want to test their milk, but allowed women who did not know the status of their breast milk to continue breastfeeding.^23^ The Table presents the above discussed recommendations.

**TABLE.** Key Breastfeeding Recommendations for Women Survivors of EVD

Date Issued	Source	Recommendation(s) or Message
August 22, 2014	ENN	“For breastfed infants of Ebola infected mothers who are asymptomatic, the risks of Ebola transmission via breastmilk are understood to outweigh the risks associated with replacement feeding.” “For breastfed infants of Ebola infected mothers who have developed Ebola or are suspected Ebola cases themselves, the benefits of maintaining breastfeeding outweigh any possible benefits of replacement feeding.” “Where a mother has survived Ebola … she should return for testing of her milk every 2–3 days (or however often is feasible) … Ideally there should be 2 negative tests on different days …”

September 2014 (update)	ENN	“If testing of breast milk is not feasible, then maternal breastfeeding should be avoided for 8 weeks post recovery.

January 2015	CDC	“Mothers who have survived Ebola should not breastfeed their infants for 2 months after recovery.”

March 2015	Social Mobilization Pillar	“If you have survived Ebola, it is best not to breastfeed IF you have other safe ways to feed your baby. But if there is no other way to feed your baby safely, breastfeeding will still provide the nutrition your baby needs.”

April 2015 (update)	CDC	(Messages consistent with Social Mobilization Pillar message above.)

April 11, 2016	WHO	“EVD survivors who are lactating may wish to have their breast milk tested … **Women who do not know the status of their breast milk or who were tested and for whom no Ebola virus RNA was detected should continue breastfeeding.** If Ebola virus RNA is detected, breastfeeding should be suspended and the breast milk restested every 48 hours until two consecutive ‘undected’ results are obtained. During this time, breast milk should be replaced with a sustainable appropriate breast-milk substitute.”

Abbreviations: CDC, U.S. Centers for Disease Control and Prevention; ENN, Emergency Nutrition Network; EVD, Ebola virus disease; RNA, ribonucleic acid; WHO, World Health Organization.

Interim clinical guidance from WHO was the most permissive, suggesting lactating women may want to test their milk but allowing women who did not know the status of their breast milk to continue breastfeeding.

## PROGRAM RESPONSE

At the same time, the MOHS and UNICEF also launched a programmatic response to address infant feeding needs. The recommendation that survivors cease breastfeeding indefinitely created a need for additional feeding options for infant children of survivors. Children under 2 years who had been orphaned or separated from their mothers were often also in need of additional feeding options. The MOHS and UNICEF responded by providing RUIF to children 6 months old or younger of women survivors. RUIF was available at the District Health Management Team (DHMT) offices in each district, and the district nutrition teams managed distribution based on demand. Although not officially included in the program, a number of orphans and separated children 6 months old or younger who had been affected by EVD also received RUIF. Children over 6 months were not eligible for inclusion as RUIF is not appropriate for this age group and other appropriate products were not available. While wet nursing was discussed, it was not considered a viable option and was not recommended by any guidelines.

This programmatic response presented a dilemma for the country at a national and district level. The national Food and Nutrition Directorate as well as the district medical officers were concerned about meeting the nutritional needs of children affected by EVD while avoiding a reduction in breastfeeding rates for non-infected women.

During the outbreak, EVD survivors received certificates upon discharge from ETCs in order to allow them to access a range of services. Survivors were also recruited for jobs that presented an infection risk to others, due to their assumed limited susceptibility to reinfection. The unique services and opportunities available to survivors inadvertently led to a demand for fraudulent survivor certificates.^24^ This posed a risk for the RUIF program, in that if the program was advertised publicly there would likely be a number of fraudulent requests for RUIF. This could contribute to a reduction in breastfeeding by women with no exposure to the Ebola virus, which would slow the progress made in optimal breastfeeding in the country. As such, the DHMT nutrition teams relied primarily on referrals from frontline health workers and civil society organizations to identify women and children who qualified for the program.

The RUIF program had 2 challenges: if the program was advertised publicly there would likely be a number of fraudulent requests for RUIF, which could lead to uninfected women requesting RUIF and a country-wide reduction in optimal breastfeeding.

## CHALLENGES

The emergence of EVD in a country with weak infrastructure and limited services for child health, as well as an emerging national breastfeeding promotion program, created a very difficult environment for decision making on infant feeding policy and programming.

### Lack of Necessary Information to Make Recommendations

As discussed, there was minimal evidence prior to the epidemic on the potential risk of EVD transmission through breastfeeding, especially in the case of survivors during convalescence. As more information emerged, policy makers were able to consider and incorporate it into relevant guidance and messaging. In some cases, such as the initial CDC messaging advising that breastfeeding could continue after 2 months, emerging information often necessitated the retraction and correction of previous health messages.

### Numerous Technical Partners

Emergency responses require participation from a large number of technical partners with different backgrounds and expertise, which can result in challenges with coordinating efforts and delineating lines of responsibility for decision making. While WHO traditionally takes the lead during an outbreak, the early months of the EVD outbreak were extremely chaotic and fast-moving and the focus of the response was primarily on immediate treatment and prevention. Recognizing the gap in infant feeding guidance, the ENN and WHO responded quickly to address that need.

### Difficulty Maintaining Communication Between Response Pillars

The pillar system offers distinct advantages for emergency response because it allows technical partners to stay informed about progress and activities in their focus area. However, when crosscutting issues emerge, the pillar system can become a barrier if communication between pillars is limited, as it was in Sierra Leone. In this case, increased communication between the Psychosocial Pillar, where the CDC EVD Survivor Information Book was reviewed, and the Social Mobilization Pillar could have prevented the dissemination of contradictory messages for EVD survivors.

### Distrust and Rumors

Behavior change in Sierra Leone is complicated due to the widespread distrust by the general population of the health system.^25^ During the EVD outbreak, this distrust was further heightened by countless rumors surrounding EVD.^25^ Messages that were disseminated and then retracted may have reinforced ideas that the government and other technical partners were incompetent or acting in their own self-interest.

## LESSONS LEARNED

Policies related to nutrition and infant feeding are ideally developed over a long period in order to allow sufficient time for collecting evidence and gaining consensus among key stakeholders. In emergency contexts, that is not always possible because of the need for a rapid response. Often, there is no other choice than to use the best information and resources available to make decisions that can be reasonably expected to have a positive impact. From this standpoint, the lessons learned from the EVD outbreak are useful in order to inform emergency preparedness planning. The recommendations from this section are summarized in the Box.

Box **Key Recommendations to Improve Responses to Future Outbreaks**

- Include infant and young child feeding experts from the beginning of the outbreak response
- Develop a digital repository for national policies to reduce conflicting messages and a clear strategy for health communication
- Prioritize research on potential vertical transmission of EVD

### Include Infant and Young Child Feeding Experts From the Beginning of the Outbreak Response

It is important that rapid response mechanisms include policy and messaging guidance development as infectious disease outbreaks become more common. These mechanisms should be headed by WHO and include country, regional, and global stakeholders. In future infectious disease outbreaks, infant and young child feeding experts should be included from the beginning of the response to ensure that nutrition issues are taken into account early on. Inclusion of these experts will also lead to faster development of relevant policies, messaging, and programming. It is also important to consider the need for appropriate breastfeeding messages for women who contract an infection while pregnant as well as those who contract an infection following birth but while still breastfeeding.

The inclusion of infant and young child feeding experts from the beginning of the response will lead to faster development of relevant policies, messaging, and programming.

### Develop a Digital Repository for National Policies to Reduce Conflicting Messages and a Clear Strategy for Health Communication

Limited communication between technical partners with different foci has been identified as a challenge in this case. A well-maintained digital repository for national policies and associated messages would improve the ability of stakeholders to ensure consistency within the response. Additionally, a clear strategy for public service communication will also increase consistency and the effectiveness of health communication. Finally, it is important for countries to have a central list of technical partners that should be made aware of the existence of such tools.

### Prioritize Research on Potential Vertical Transmission of Ebola Virus Disease

One of the major challenges to developing infant feeding guidance for EVD survivors was the limited information on the potential risk of vertical transmission during the acute and the convalescent phases of EVD through breast milk and other paths. Although the outbreak is now over, those risk pathways are still poorly understood. The thousands of survivors in Sierra Leone and other affected West African countries could provide countries a unique opportunity to increase understanding of the virus's short- and long-term effects on the human body. It is vital that a better understanding of the potential risk of vertical transmission through breast milk is achieved in order to improve the breastfeeding policy response in future Ebola outbreaks.

One of the major challenges to developing infant feeding guidance for EVD survivors was the limited information on the potential risk of vertical transmission during the acute and the convalescent phases of EVD.

## PARALLELS TO HIV POLICY DEVELOPMENT

While there is now a large evidence base to draw from when making breastfeeding policy and programming recommendations for mothers with HIV,^26^ this was not the case in the early years of the HIV epidemic.^27^ It is not surprising that global and country-level guidance on breastfeeding by HIV-infected mothers has continuously evolved throughout the past several decades as new disease knowledge and prevention and treatment options emerged.^27^ The HIV example provides insight into how guidance to reduce the risk of vertical transmission through breastfeeding should evolve as new evidence improves understanding. Acute outbreaks offer the opportunity to better understand how long-term international policy-making processes can be adapted to an emergency response within an accelerated period.

Acute outbreaks offer the opportunity to better understand how long-term international policy-making processes can be adapted to an emergency response within an accelerated period

## CONCLUSION

In examining this Field Action Report, it is important to bear in mind the challenges of the environment in Sierra Leone during the EVD outbreak. Actors on the ground were faced with innumerable competing demands on their time and attention and were hindered by limited information about the potential risk of transmission through breastfeeding. The evolution of the messages that emerged during this period is reflective of the priorities and available information at different points in the outbreak. Earlier messages referred to a 2-month period after recovery when breastfeeding should be suspended, but as more evidence emerged about the length of viral persistence in breast milk, the messages evolved to be less specific regarding time frames. These messages also evolved to be more permissive, allowing that if no other safe way to feed the infant existed that breastfeeding was still the best option. This was evidenced by the final messages released by WHO after the end of the outbreak, which prioritized the well-documented benefits of breastfeeding over the risk of transmission, which, at that point seemed to be no longer a concern.

Intersections between infectious disease outbreaks and maternal and child health policies are becoming increasingly important. While this report focuses on the intersection of infant feeding policy in the case of the EVD outbreak, other examples are emerging, such as health policies related to the ongoing Zika epidemic. Public health practitioners focused on infectious diseases and maternal and child health have not always worked closely together, but there is a growing need to consider the links between them. The EVD outbreak in West Africa provides a case study of the nature of these links and can provide insight on the importance of working together to limit setbacks in maternal and child health due to infectious disease outbreaks.

## Notes

**Peer Reviewed**
